# Editorial: Innovation in diabetes self–care management and interventions

**DOI:** 10.3389/fendo.2023.1269437

**Published:** 2023-08-21

**Authors:** Edward Zimbudzi, Hiroshi Okada, Martha M. Funnell, Masahide Hamaguchi

**Affiliations:** ^1^ School of Nursing and Midwifery, Monash University, Melbourne, VIC, Australia; ^2^ Department of Nephrology, Monash Health, Melbourne, VIC, Australia; ^3^ School of Public Health and Preventive Medicine, Monash University, Melbourne, VIC, Australia; ^4^ Department of Endocrinology and Metabolism, Graduate School of Medical Science, Kyoto Prefectural University of Medicine, Kyoto, Japan; ^5^ Department of Learning Health Sciences, University of Michigan Medical School, Ann Arbor, MI, United States

**Keywords:** self-management, innovation, diabetes complications, technology, patient-centered interventions

In recent years, the number of patients with type 2 diabetes mellitus (T2DM) has increased. In 2021, it was estimated that 537 million adults worldwide were living with diabetes with the total number projected to rise to 643 million by 2030 (1). In view of this, new innovations including person-centered self-management interventions are needed to prevent new onset of diabetes and the development of complications associated with diabetes. Over the past two decades, a significant number of diabetes self‐management education and support programmes have been developed and translated into practice (2). Multiple studies and meta-analyses have shown that these programmes are efficacious and cost‐effective in promoting and facilitating self‐management and improvements in patients’ knowledge, biomedical, behavioural, and psychosocial outcomes have been reported (3–7). Among this array of diabetes self‐management education programmes, variations in method of delivery, content, duration, setting, and use of technology and person‐centred philosophy need to be acknowledged (2). Most importantly, questions need to be asked regarding the usefulness of some programmes, which are anchored on the traditional provider–patient relationship due to a huge shift towards a paradigm in which individuals with diabetes play a key role in guiding their care, in partnership with health care providers. Within this context, we launched our Research Topic on April 7th, 2022, and invited researchers to submit articles that explore *Innovations and Interventions in Diabetes Self‐Management and Interventions.*

This Research Topic reports on new insights, challenges, and future directions regarding innovations in diabetes self-management. The Research Topic generated a lot of interest across a broad range of critical issues resulting in the publication of 13 articles (all in Frontiers in Endocrinology), involving 113 authors from 7 countries. These articles covered the following themes; (1) use of technology in managing complications of diabetes and (2) novel approaches to optimise diagnosis, monitoring and self-management interventions for people with diabetes.

A first line of research includes contributions examining the use of technology in addressing complications associated with diabetes. One of the complications of diabetes is Erectile Dysfunction (ED) which affects over two-thirds of men (8), and this is normally treated with phosphodiesterase type 5-inhibitors (PDE5is). However, a substantial number of people with ED do not respond to PDE5is necessitating the use of other therapies. Tao et al. show that the combined therapy of low intensity extracorporeal shock wave treatment (Li-ESWT) and vacuum erectile device (VED) is more beneficial to shift turn PDE5is non-responders to responders for moderate impotence men with diabetes than Li-ESWT or VED monotherapy due to their synergistic effect. Diabetic foot ulcers (DFU) are also a well-recognised complication of diabetes. Sousa et al. report on a protocol of a study that aims to develop innovative footwear to prevent DFU, specifically a shoe and sensor-based insole, which will allow for monitoring pressure, temperature, and humidity parameters.

A second stream of research includes studies that focus on novel approaches to optimise diagnosis, monitoring and self-management interventions for people with diabetes. Two studies from Sun et al. and Byeon et al. report on nomograms that can be used to optimize screening of diabetes mellitus in people at risk of diabetes. These two studies address a very important concept that may result in the reduction of the lead time between diabetes onset and clinical diagnosis allowing for prompt multifactorial treatment to be initiated if warranted. Another study established a model using fasting capillary blood glucose (FCG) and postprandial capillary blood glucose (PCG) together to predict HbA1c in patients with T2DM (Yuan et al.). This approach provides an available and convenient way to convert real-time SMBG readings to HbA1c resulting in timely management by people with T2DM. While it is recognised that SMBG is one of the pillars of diabetes management for patients with diabetes, we need to better support our patients in implementing and using the data for daily decision-making from SMBG (9). In relation to this, Lin et al. described the current status of SMBG among pre-diabetes patients and those with T2DM, explored the relationship between SMBG frequency and blood glucose level and analyzed the potential factors that influence patient implementation and use of SMBG based on electronic questionnaires using the information-motivation-behavior model.

In prediabetes, it is known that weight loss can delay the onset or decrease the risk for T2DM, while in established T2DM weight loss improves glycaemic control, with severe calorie restriction even reversing the progression of T2DM (10). In view of this, a study by Almeida et al. show that a technology-enhanced diabetes prevention program is effective in reducing body mass index at 6 months and maintaining these results at 12 and 18 months in a group of primary care patients at risk for developing T2DM. Matsui et al. investigated the association between change in body weight and T2DM remission in Japanese men with new-onset T2DM. A weight loss of ≥5% effectively achieved diabetes remission for those with a BMI ≥25 kg/m^2^ and new-onset T2DM.

Exercise is one of the first management approaches advised for patients newly diagnosed with T2DM. Matsushita et al. investigated the effects of physical therapists’ exercise instructions among Japanese patients with T2DM. After 8 weeks of follow up, HbA1c levels were significantly better in the intervention group than among the non-intervention group (7.3% [6.8-%–7.9%] vs. 7.4% [7.3%–7.7%], P = 0.04). Additionally, the intervention group had more improved motor skills than the non-intervention group, and the transtheoretical model varied in the intervention group but not in the non-intervention group between before and after intervention.

Feng et al. conducted a randomized double-blind placebo-controlled trial to explore the effects of a highly active α-amylase inhibitor derived from white common bean extract (WCBE) on glucose metabolism and diabetes complications in patients with T2DM. There was a greater reduction in HbA1c levels among patients who received the WCBE compared to those who did not at the end of the 2-month intense intervention (0.660 ± 0.468% vs. 0.222 ± 0.763%, p<0.05) and at the end of the second 2-month intervention (0.721 ± 0.742% vs. 1.059×10-8 ± 0.942%, p<0.05) suggesting that using this dietary supplement can potentially lower HbA1c levels. The proportion of patients with diabetic peripheral neuropathy (measured by the Toronto Clinical Scoring System, TCSS ≥ 6) was significantly lower in the intervention group compared to the control group. Additionally, both the left and right sural sensory nerve conduction velocity slightly decreased among those in the control group and slightly increased in the intervention group suggesting that the intervention may potentially improve complications such as diabetic vasculopathy and neuropathy.

A systematic review from Racey et al. explored the effects of health coaching with adults with T2DM based on patient-reported outcomes, clinical outcomes, provider satisfaction, and cost-effectiveness. Findings from this study suggest that health coaching interventions can have short term impact beyond glucose management on cardiometabolic and mental health outcomes. In another systematic review (Racey et al.), the same authors examined aspects of diabetes health coaching interventions for adults living with T2DM that have been reported using the Reach, Effectiveness, Adoption, Implementation, and Maintenance (RE-AIM) framework to optimize implementation. They found that there is paucity of reporting of the RE-AIM components for diabetes health coaching leading to limited implementation and clinical practice implications that can be drawn.

A study from Moghaddam et al. examined the determinants of quality of life among elderly patients based on problem areas in diabetes (PAID). As expected, they report that treatment barriers, psychological distress related to the burden of diabetes, the type of treatment, and age had a negative impact on the quality of life of elderly patients. Findings from this study reinforce the importance of considering diabetes-specific distress, treatment barriers and patient barriers and preferences when discussing interventions.

Overall, these findings highlight that patient-centered interventions to support self-management of diabetes and its complications are evolving. Notably, it is encouraging to see that a number of these interventions embrace the use of technology. The strengths of these contributions include the use of randomised control trials and systematic reviews and meta-analyses to examine the effect of interventions on outcomes. However, results from some studies may not be generalisable due to the inclusion of people whose BMI was lower than that of other populations. Additionally, the long-term efficacy and safety of some interventions still needs to be ascertained. Nevertheless, we are confident that all the selected studies in our Research Topic bring important perspectives to the understanding of current innovations in diabetes self-management and person-centered interventions.

## Author contributions

EZ: Writing – original draft, Writing – review & editing. HO: Writing – review & editing. MF: Writing – review & editing. MH: Writing – review & editing.
